# Theoretical Study on Adsorption of Halogenated Benzenes on Montmorillonites Modified With M(I)/M(II) Cations

**DOI:** 10.1002/jcc.70042

**Published:** 2025-01-28

**Authors:** Daniel Tunega, Martin H. Gerzabek, Leonard Böhm

**Affiliations:** ^1^ Institute for Soil Research, Department of Forest and Soil Sciences University of Natural Resources and Life Sciences Vienna Vienna Austria; ^2^ Institute of Soil Science and Soil Conservation, Research Centre for BioSystems, Land Use and Nutrition (iFZ) Justus Liebig University Giessen Giessen Germany

**Keywords:** adsorption, alkali and alkaline earth elements, density functional theory, halogenated benzenes, montmorillonite

## Abstract

Halogenated benzenes (HBs) are hydrophobic organic chemicals belonging to persistent organic pollutants. Owing to their persistence, they represent a serious problem in environmental contamination, specifically of soils and sediments. One of the most important physical processes determining the fate of HBs in soils is adsorption to main soil components such as soil organic matter and soil minerals. Smectites, layered clay minerals of the 2:1 type, are common minerals in clay‐rich soils, of which montmorillonite (Mt) is a typical representative. This work focuses on a systematic modeling study of the adsorption mechanism of selected HBs interacting with the basal (001) surface, which is the dominant surface of Mt particles. The HB···Mt interactions were studied by means of a quantum chemical approach based on the density functional theory method. HBs were represented by five molecules, particularly C_6_F_6_, C_6_Cl_3_F_3_, C_6_Cl_6_, C_6_Br_3_Cl_3_, and C_6_Br_6_. In mixed HBs (C_6_Cl_3_F_3_ and C_6_Br_3_Cl_3_) Cl atoms are in 1,3,5 or rather 2,4,6 positions. The effect of a different cation type on adsorption was investigated for M^+^/M^2+^‐Mt models with cations from alkali group (M^+^: Li, K, Na, Rb, Cs) and alkaline earth metal group (M^2+^: Mg, Ca, Sr., Ba). The calculations were also performed on the gas phase HB···M^+^/M^2+^ complexes for comparison. Adsorption energies and distances of the main HB molecular plane from the Mt surface were calculated as a measure of the adsorption strength. The results showed that the strongest HB adsorption is for the Na^+^‐Mt and Ca^2+^‐Mt surfaces. The strongest affinity was observed for hexabromobenzene, while the weakest adsorption was found for hexafluorobenzene. The decomposition of the adsorption energy showed that its dominant component is dispersion energy and less important is the cation‐π interaction. The calculated adsorption energies showed a good correlation with experimentally determined log *K*_d_ values.

## Graphical abstract

Adsorption energies of five halogenated benzenes at clay mineral montmorillonite modified with cations of alkali and alkaline earth elements were calculated by methods of density functional theory and using periodic slab models.

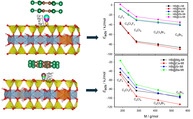

## 1 Introduction

Halogenated Benzenes (HBs) belong to a rich group of halocarbons (HCs), many of which have found industrial and agricultural use as pesticides (mainly Cl‐based HCs), refrigerants (mainly F‐based HCs), fire retarders (mainly Br‐based HCs), and solvents, as ingredients in adhesives and sealants, in electrically insulating sprays and glues, plasticizers, and plastics [1]. HCs are mostly hydrophobic organic chemicals (HOCs) frequently with a long longevity what represents a potential risk for the ecosystems and human health. Specifically, some chlorocarbons are very harmful as they were used in past widely in the agriculture as pesticides and they still exist in high concentrations in different environmental compartments such as soils, sediments, and water [2, 3, 4]. They belong to the group of persistent organic pollutants (POPs), which typically have a long life in the environment, where they can bioaccumulate in food webs due to their lipophilic and hydrophobic properties what represents a high risk for humans and animals [1, 5, 6]. Owing to their high persistence and environmental risk, there is an enormous effort to develop effective and cost‐acceptable remediation techniques for their removal from the ecosystems [7, 8].

Of the HBs, hexachlorobenzene (HCB) is an example of a POP chemical that was used extensively in the past as fungicide in agriculture. Nowadays, HCB belongs to the group “Dirty Dozen Chemicals,” which are banned worldwide by the Stockholm Convention in 2001 (United Nations Environment Programme (UNEP) 2020). Nevertheless, it is still found in various contaminated places [9, 10, 11, 12] in significant concentrations because of its high persistence. For example, HCB was also found ubiquitously in Rhine River sediments [13]. Besides its restricted status, HCB is still released to the environment, for example, from fuel combustion and the metal industry. Except HCB, also hexabromobenzene (HBB) is found in contaminated sites [14, 15, 16, 17, 18] because it was used as flame retardant in plastics, and paper to reduce combustion [19]. The fate of the HBs in soils and sediments is related to its transportation and accumulation in solid matrices and biota (less in aqueous systems due to their low solubility). Thus, these processes are linked to adsorption at interfaces of solid–aqueous systems. There are numerous papers devoted to the transportation, accumulation, adsorption, and remediation of HCB in soils and sediments, including several reviews [9, 20, 21, 22, 23]. Adsorption studies of HCB to soils and sediments include also works focusing on the adsorption to soil phases such as soil organic matter (SOM) [24, 25, 26, 27, 28, 29] and, in rare cases, also to pure minerals including clays [30, 31]. Indeed, adsorption experiments on other HBs to soil components are much less frequent comparing to HCB. For example, hexafluorobenzene (HFB) on montmorillonite was used as a probe to reveal *n* − *π* electron−donor−acceptor (EDA) mechanism in complexation of an aromatic moiety with an exchangeable cation of clay [32].

Our recent study [31] on the adsorption of HCB to a set of clay minerals showed a large variation of adsorption spanning over several orders of magnitude (log *K*_d_ 0.9–3.3). Indeed, adsorption to pure mineral phases was lower as compared with the adsorption of HCB to organic matter [6, 29]. That work also included the adsorption study to montmorillonite (STx‐1b) modified with cations of alkali and alkaline earth elements to show the impact of the cation type on the adsorption strength. Moreover, the work also included theoretical modeling (based on the density functional theory, DFT) of the interactions of HCB with the cation‐modified montmorillonite models (M(I)/M(II)‐Mt) quantifying the adsorption strength of the varying cations. That work was followed by the theoretical study of the impact of the varying layer charge (−0.125 to −0.75 |*e*|) using the HCB molecule as a probe and Ca^2+^‐Mt as a layer model [33]. It was shown that the increasing layer charge had only a minimal impact on the adsorption strength of the HCB molecule.

Generally, there are only a few theoretical studies on interactions and adsorption of halogenated benzenes with solid surfaces. Adsorption of several organochlorine pollutants (including HCB) on different types of zeolites was modeled by using Grand Canonical Monte Carlo approach (based on classical force field parameters) to show a shape selectivity adsorption [34]. The photodegradation mechanism of HCB at the surfaces of ZnCr layered double hydroxides was elucidated by using DFT approach [35]. DFT was also used in the study of self‐assembling of fully halo‐substituted benzenes C_6_X_6_ on the Ag (110) surface [36]. Dispersion‐corrected DFT method was also applied in the explanation of the effect of the sigma hole interaction in the adsorption of a set of halobenzenes on the (111) Cu surface [37]. Competition between hexagonal and tetragonal packing of the HBB molecule at the Au(111) surface was studied both experimentally and theoretically (DFT) [38]. DFT study was also used in the explanation of the effect of silica gel surface on the HCB degradation [39]. The exchangeable cations are expected to play a significant role in the adsorption process of (poly)aromatic hydrocarbons and their halogenated derivatives on mineral surfaces. Several theoretical works were devoted to interactions of ions with aromatic systems [40, 41, 42, 43, 44]. However, a systematic theoretical study of the adsorption of halogenated aromatic species on clay mineral surfaces is still missing.

This work is aimed to explain how the adsorption of five fully halogenated benzenes (C_6_F_6_, C_6_Cl_3_F_3_, C_6_Cl_6_, C_6_Br_3_Cl_3_, and C_6_Br_6_) is affected by the type of the cation compensating layer charge in montmorillonites. For this purpose, layer models of montmorillonite with different compensating cations of alkali and alkaline earth elements (M^+^: Li, K, Na, Rb, Cs; M^2+^: Mg, Ca, Sr., Ba) were used in the calculation of adsorption energies of five HBs molecules by using DFT method. In addition to energies, geometrical parameters were discussed as well. The results for HB···M(I)/M(II)‐Mt complexes were compared with pure HB···cation complexes in order to determine the origin of the interactions and to quantify the effect of the Mt layer on the adsorption.

## 2 Computational Details

### 2.1 DFT Calculations

The calculations were performed by the Vienna Ab initio Simulation Package (VASP) [45, 46, 47, 48] using the Perdew–Burke–Ernzerhof (PBE) functional [49] and plane wave basis set with energy cutoff of 400 eV. The projector‐augmented wave atomic pseudopotentials (PAW) was applied for describing electronic structure of atoms [50, 51]. In addition, D3 type of dispersion corrections was included in calculations [52]. The *k* point sampling was restricted to *Γ* point as large computational cells were used for models. The electronic energy convergence was set to 10^−4^ eV, and for the geometry optimization a force convergence criterion of 0.01 eV/Å was applied. The adsorption energy of the HB molecules, *E*_ads_, was calculated as a difference between the total energy of the optimized HB···Mt adsorption complex and a sum of the total energies of the optimized isolated Mt layer and the isolated HB molecule (Equation 1). In both isolated cases the same computational cell as for the HB···Mt complex was used.

(1) $$E_{\mathrm{ads}}=E\left(\text{HBMt}\right)-\left(E\left(\mathrm{Mt}\right)+E\left(\mathrm{HB}\right)\right)$$

In addition, calculations on the HB molecules and the gas phase HB···cation complexes were performed with the program TURBOMOLE [53, 54] using the same PBE‐D3 functional as in the VASP calculations, the Resolution of Identity (RI) approach [55], and large atomic basis set of the def2‐TZVP quality [56, 57]. In these calculations, we also used the polarizable continuum method representing water solvent of the COSMO type [58] to achieve some physical parameters of the HB molecules such as molecular volume and molecular surface area that represent a cavity, in which the HB molecule is embedded in the COSMO calculation.

### 2.2 HBs Molecules and M(I)/M(II) Models

**FIGURE 1:**
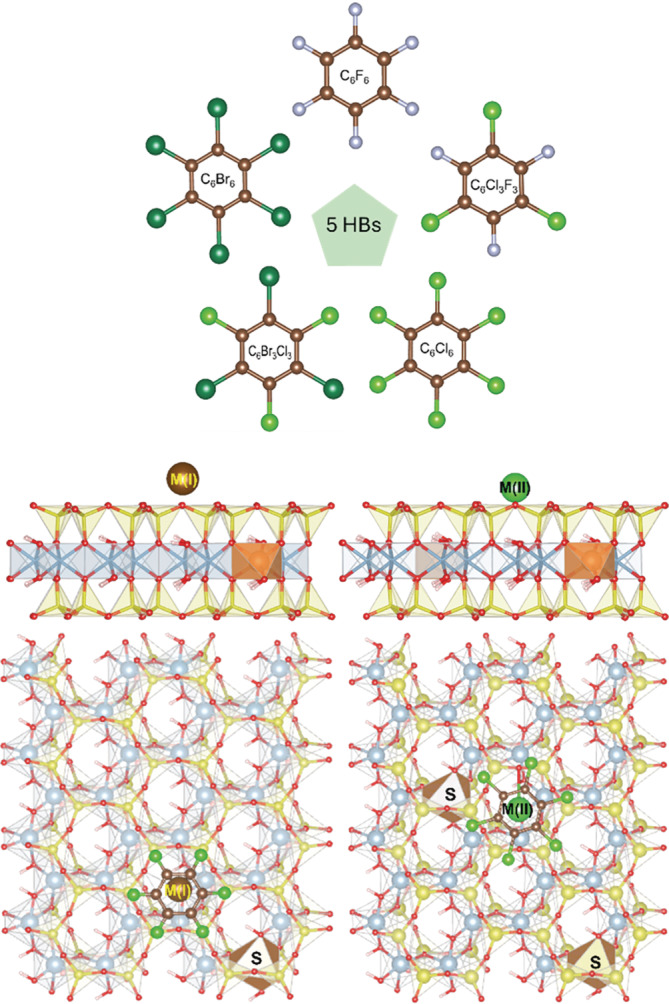
Structures of five HB molecules and models of M(I)‐Mt (left) and M(II)‐Mt (right) layers. Side and top views on Mt models are displayed. “S” represents the position of Al^3+^/Mg^2+^ substitutions in the octahedral sheet.

Five HB molecules were investigated in this study, particularly C_6_F_6_ (hexafluorobenzene, HFB), C_6_Cl_3_F_3_ (1,3,5‐trichloro‐2,4,6‐trifluorobenzene, TCTFB), C_6_Cl_6_ (hexachlorobenzene, HCB), C_6_Br_3_Cl_3_ (1,3,5‐tribromo‐2,4,6‐trichlorobenzene, TBTCB), and C_6_Br_6_ (hexabromobenzene, HBB). In the mixed HBs (C_6_Cl_3_F_3_ and C_6_Br_3_Cl_3_), Cl atoms are symmetrically substituted in 1,3,5 or rather 2,4,6 positions (Figure 1). Structural models representing one montmorillonite layer (Mt) with one (for alkali metal cations) or two (for alkaline earth metal cations) octahedral Al^3+^/Mg^2+^ substitutions (Figure 1) were the same as used in our previous paper [31] to be compatible with the calculations for the HCB molecule in that paper. The substitutions in the octahedral layer resulted in formulas M(I)_0.125_(Al_3.875_Mg_0.125_)(Si_8_O_20_)(OH)_4_ and M(II)_0.125_(Al_3.75_Mg_0.25_)(Si_8_O_20_)(OH)_4_. The calculations were performed using periodic slab models with lateral *a* and *b* lattice vectors of 20.97 and 18.18 Å, respectively. In the third lattice dimension, a 20 Å vacuum was added to minimize interactions between periodic images in the *c* direction. These lattice parameters were achieved in our previous work [31] by the optimization of the computational cell of the Na^+^‐Mt single layer and were also used for the models with other cations. In all calculations performed in this work, the lattice vectors were fixed during the geometry optimization to have the same reference computational cell.

The used Mt model offers 16 potential positions for the M(I)/M(II) compensating cations being represented by ditrigonal holes. The permanent negative layer charge is produced by the cation substitution only in the octahedral sheet. Therefore, this charge is delocalized over all surface oxygen atoms as was evidenced by the calculated Bader atomic charges [59, 60], which are in narrow ranges −1.562 to −1.587 |*e*| (Na‐Mt model), and −1.556 to −1.585 |*e*| (Ca‐Mt model), respectively. Thus, we supposed that the choice of a particular position for the compensating cation is not an important factor, and using other sites would have little effect on the calculated data in the form of a systematic shift. Therefore, in the initial geometries of the M(I)/M(II)‐Mt models, the compensating cations were placed above a center of one selected ditrigonal hole in the tetrahedral sheet as shown in Figure 1. The initial position of the HB molecule was in a parallel configuration of the main HB plane with the Mt surface and with a center of the molecule above the compensating cation. Then, all atomic positions were optimized keeping the computational cell fixed.

## 3 Results and Discussion

In our previous work [31], we focused on the explanation how montmorillonites with different compensating cations from groups of alkali elements (M(I): Li^+^, Na^+^, K^+^, Rb^+^, Cs^+^) and alkaline earth elements (M(II): Mg^2+^, Ca^2+^, Sr^2+^, Ba^2+^) adsorb the HCB molecule. The calculated adsorption energies, *E*_ads_, were related to several physical parameters of cations such as ionic radius (*IR*), hydration enthalpy, and/or ionic charge density. The work also related the calculated *E*_ads_ with experimentally determined log*K*_d_ values (adsorption constants, *K*_d_, were obtained from the fit of Henry adsorption isotherm to experimental data) for HCB adsorbed on homoionically modified STx‐1b montmorillonite with nine alkali and alkaline earth exchangeable cations.

This theoretical work focused on the gas phase adsorption of five HB molecules (Figure 1) on the nine M(I)/M(II)‐Mt one‐layer models. The main physical parameters of five HBs are collected in Table 1. Molecular volume (*V*) and molecular surface area (*A*) were obtained from the PBE‐D3 COSMO calculations and represent a cavity in which the HB molecule is embedded. Distances X—X and C—C are from the optimized COSMO geometries. Generally, HB molecules are considered as hydrophobic molecules with low solubility, small hydration energies, and large *n*‐octanol/water partitioning coefficients (log*K*_ow_ in a range 4–6) [61]. The calculated COSMO free solvation energies, *G*_sol_ (Table 1), confirm the hydrophobic character of HBs, even if in some cases they differ from the experimental values (e.g., −11.78 kJ/mol in Table 1 versus − 17.1 kJ/mol from Ref. [61]). The size of the HB molecule is dictated by the type of the halogen atom, particularly by the C—X bond and the X atomic radius. Evidently, the largest molecule is HBB while the smallest one is HFB (Table 1). However, according to the C—C distance, the size of the aromatic ring changes only minimally. The difference between C_6_F_6_ and C_6_Br_6_ in the C—C distance is only about 0.02 Å (Table 1).

**TABLE 1:** Molecular mass (*M*), molecular volume (*V*), molecular surface area (*A*), and diagonal atom‐to‐atom distances (X—X is halogen to halogen atom distance), and solvation free energy (*G*_sol_) of five HBs molecules. *V*, *A*, X—X, C—C, and *G*_sol_ are obtained from optimized geometries in COSMO calculations.

	*M*/Da	*V*/Å [3]	*A*/Å [2]	X—X/Å	C—C/Å	*G*_sol_/kJ/mol
C_6_F_6_	186.06	158.88	162.80	5.474	2.791	−9.39
C_6_Cl_3_F_3_	235.41	199.57	193.75	5.854	2.796	−8.88
C_6_Cl_6_	284.78	237.33	219.56	6.254	2.811	−8.08
C_6_Br_3_Cl_3_	418.13	258.38	233.21	6.422	2.812	−9.90
C_6_Br_6_	551.49	275.53	245.01	6.597	2.813	−11.78

**FIGURE 2:**
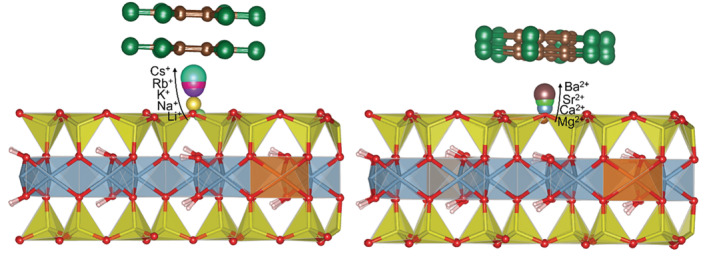
Superposition of the optimized geometries for all five HBB···M(I)‐Mt adsorption complexes (left), and for all four HBB···M(II)‐Mt adsorption complexes (right).

The Mt layer models represent the most dominant basal surface of montmorillonite particles, (001) (Figure 1). This study does not include other potential models for the adsorbed HB molecules such as intercalated molecules in the interlayer space or on the broken edges of Mt layers. Our previous papers [31, 33] showed that the most stable configuration for the adsorbed HCB molecule is in a planar configuration (with the molecular plane parallel to the basal surface) and with the center of the aromatic ring above the compensating cation. This configuration was also used for the other four HB molecules in this study. The results of the geometry optimizations for all models are graphically displayed in Figure 2. It shows the superposition of the geometries of the adsorbed C_6_Br_6_ molecule of all five M(I)‐Mt models (left) and of all four M(II)‐Mt models (right).

The main geometry parameters (perpendicular distances of the molecular plane to the cation, *d1*, and of the cation to the plane of the basal surface oxygen atoms of the Mt layer, *d2*) are collected in Table 2, and the calculated adsorption energies and are shown in Table 3. For comparison, we also provide results for the pure HB–M(I)/M(II) gas phase complexes, without the presence of the Mt layer. These molecular complexes were optimized at the PBE‐D3/def2‐TZVP level. The complex formation energies (*E*_cf_) are collected in Table 3 and the corresponding perpendicular distances of the cation to the HB molecular plane (*d1*_g_) are presented in Table 2. This table also contains ionic radius of all nine cations. *IR* is not an exactly defined parameter and depends on the chemical environment of the particular ion. Therefore, we decided to take *IR* as defined for ions in aqueous solvent [62].

**TABLE 2:** Ionic radii (*IR*) of M(I) and M(II) cations (in Å), HB–cation distances (*d1*_g_ in gas phase complexes, *d1* in adsorption complexes of HB at M(I)/M(II)‐Mt layer), and *d2* (distance between cation and plane of basal oxygen atoms of the Mt layer). All distances are in Å.

M(I)/M(II)	*IR* ^*a*^	C_6_F_6_			C_6_Cl_3_F_3_			C_6_Cl_6_			C_6_Br_3_Cl_3_			C_6_Br_6_		
		*d1*_g_	*d1*	*d2*	*d1*_g_	*d1*	*d2*	*d1*_g_	*d1*	*d2*	*d1*_g_	*d1*	*d2*	*d1*_g_	*d1*	*d2*
Li	0.71	1.978	3.433	−0.100	1.930	3.429	−0.094	1.890	3.411	−0.077	1.878	3.412	−0.079	1.864	3.412	−0.078
Na	0.97	2.633	2.962	0.467	2.534	2.958	0.472	2.462	2.956	0.482	2.440	2.955	0.483	2.417	2.950	0.488
K	1.41	3.250	3.739	1.367	3.098	3.734	1.371	2.959	3.727	1.383	2.919	3.726	1.383	2.894	3.724	1.385
Rb	1.50	3.504	3.649	1.590	3.380	3.648	1.592	3.215	3.645	1.599	3.171	3.643	1.601	3.131	3.639	1.605
Cs	1.73	3.744	3.442	1.827	3.546	3.432	1.837	3.357	3.417	1.856	3.318	3.413	1.861	3.283	3.410	1.865
Mg	0.70	2.037	3.267	−0.179	1.976	3.256	−0.172	1.921	3.230	−0.154	1.903	3.230	−0.155	1.886	3.229	−0.153
Ca	1.03	2.420	3.069	0.258	2.346	3.041	0.294	2.291	3.026	0.311	2.273	3.015	0.317	2.258	3.005	0.324
Sr	1.25	2.652	3.245	0.621	2.566	3.236	0.685	2.504	3.222	0.701	2.484	3.170	0.702	2.467	3.165	0.736
Ba^b^	1.35	2.845	3.125	0.930	2.757	3.063	0.990	2.694	3.027	1.026	2.675	3.019	1.035	2.645	3.012	1.042

^a^ From Marcus (1988) [61].

^b^ From Shannon (1976) [63].

**TABLE 3:** PBE‐D3 calculated complex formation energies for HB···cation complexes (*E*_cf_), and adsorption energies for HB molecules at M(I)/M(II)‐Mt surfaces (*E*_ads_). Energies are in kJ/mol.

M(I)/M(II)	C_6_F_6_		C_6_Cl_3_F_3_		C_6_Cl_6_		C_6_Br_3_Cl_3_		C_6_Br_6_	
	*E*_*c*f_	*E*_ads_	*E*_*c*f_	*E*_ads_	*E*_*c*f_	*E*_ads_	*E*_*c*f_	*E*_ads_	*E*_*c*f_	*E*_ads_
Li	−54.15	−38.69	−77.61	−57.24	−99.98	−73.98	−108.47	−79.77	−116.81	−86.44
Na	−22.72	−35.67	−41.59	−56.42	−61.0	−75.94	−68.54	−82.59	−76.07	−90.16
K	−11.51	−8.20	−27.32	−19.80	−44.1	−31.07	−50.76	−35.87	−57.47	−40.97
Rb	−10.20	−7.29	−25.01	−18.99	−40.7	−29.75	−50.76	−34.57	−52.82	−39.11
Cs	−8.62	1.09	−22.89	−11.90	−38.7	−23.83	−44.94	−29.55	−51.29	−35.11
Mg	−330.43	−43.18	−400.90	−64.66	−461.5	−83.72	−486.48	−90.15	−510.33	−96.74
Ca	−205.98	−48.15	−268.15	−72.48	−323.8	−96.19	−348.71	−104.90	−372.29	−114.15
Sr	−143.45	−34.58	−199.00	−54.44	−250.6	−76.48	−273.83	−89.22	−295.97	−99.13
Ba	−114.61	−16.65	−166.91	−43.86	−215.5	−70.49	−237.23	−82.30	−222.65	−93.74

**FIGURE 3:**
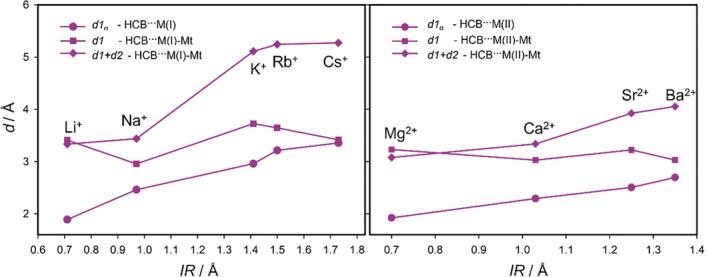
Relation between perpendicular HCB–cation distance and ionic radius (*IR*) of M(I) (left) and M(II) (right). *d1*_g_ is the distance in gas phase complexes, *d1* in HCB···M(I)/M(II)‐Mt adsorption complexes, and *d1* + *d2* is the distance of HCB to the plane of basal oxygen atoms of Mt surface.

HB–cation distances for the gas phase complexes (*d1*_*g*_ in Table 2) span in a range ~2.0 to ~3.8 Å for the M(I) cations and in a range ~2.0 to ~2.9 Å for the M(II) cations, respectively. They monotonically increase with the increasing size of the cation (IR). The results for the HCB molecule are shown graphically in Figure 3. Cations of the M(I) elements can be separated into two groups—cations with the small *IR* (Li^+^ and Na^+^), and with the large *IR* (K^+^, Rb^+^, and Cs^+^). Such separation is not evident for the cations of the M(II) elements. Moreover, ionic radii of the large M(I) cations are larger than *IR* of the Sr^2+^ and Ba^2+^ cations. *IR* of Li^+^ and Na^+^ are similar to *IR* of Mg^2+^ and Ca^2+^ that is also reflected in the similar *d1*_g_ distances for Li^+^/Mg^2+^, and Na^+^/Ca^2+^ pairs (Table 2).

*IR* is also a driving factor determining the position of the HB molecules in the adsorbed HB···M(I)/M(II)‐Mt systems. For small cations, the distance between the HB molecule and cation (*d1*) significantly increases comparing to the *d1*_g_ (see example for HCB molecule in Figure 3). This is due to the fact that the perpendicular distances of small cations to the plane of basal oxygen atoms of montmorillonite layer (*d2* in Table 2) are small. Even the smallest cations Li^+^ and Mg^2+^ are embedded in the ditrigonal hole of the basal oxygen atoms (negative *d2* values in Table 2). If the HB–cation distances in the adsorption complexes would be similar to those in the gas phase complexes, then the HB molecules would be too close to the basal oxygen atoms. Thus, the repulsion between the HB molecule (mainly of halogen atoms) and the basal oxygen atoms shifts the HB molecule farer from the cation. For the large cations, this effect is less evident and, for example, for the Cs^+^ cation, the HB–cation distance *d1*_g_ is similar to the *d1* distance (Figure 3).

**FIGURE 4:**
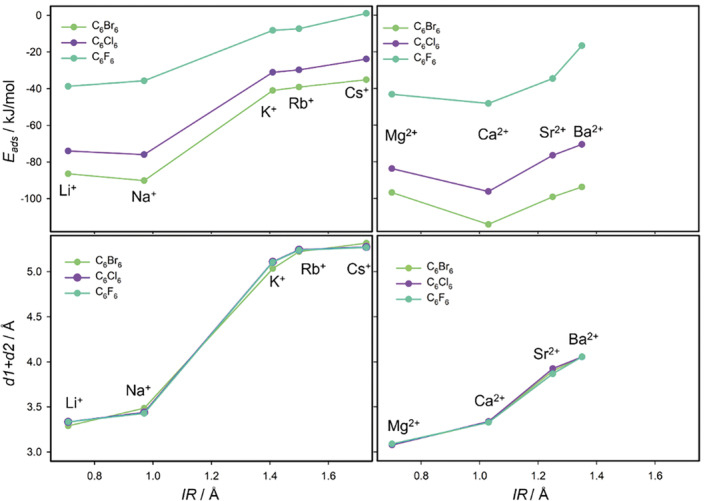
Dependences of adsorption energies (*E*_ads_) and total perpendicular distances (*d1* + *d2*) for three HBs molecules in HB···M(I)/M(II)‐Mt adsorption complexes on ionic radius (*IR*) of alkali element cations (left) and alkaline earth cations (right).

The sum *d1* + *d2* represents the perpendicular distance of the HB molecule to the plane of the basal oxygen atoms. This distance monotonically increases with the increasing size of the cations, more significantly for the M(I) cations (see graphical example for the HCB molecule in Figure 3 and two bottom plots in Figure 4). Moreover, the separation on small and large M(I) cations is evident from the *d1* + *d2* distances what is also visible in Figure 2 displaying the superposition of optimized geometries for the C_6_Br_6_ molecule and all M(I) cations. For the M(II) cations such separation is not visible (Figure 2). The similarity between *IR* of the small M(I) cations and *IR* of Mg^2+/^Ca^2+^ is also reflected in the similar *d1* + *d2* distances. However, for the larger cations from the same row of the periodic table (K^+^/Sr^2+^ and Rb^+^/Ba^2+^) overall distances differ quite a lot (distances for Sr^2+^/Ba^2+^ are about 4 Å, while for K^+^/Rb^+^ are more than 5 Å) what is a consequence of different *IR*.

Comparison of the perpendicular distances for all HB molecules to the Mt layer showed only minimal differences as it is shown in two bottom plots in Figure 4 for C_6_F_6_, C_6_Cl_6_, and C_6_Br_6_.

In contrast to the HB–Mt distances, calculated adsorption energies (*E*_ads_) differ significantly (Table 3). This table also contains complex formation energies (*E*_cf_) of the gas phase HB···MI(I)/M(II) complexes. Large *E*_cf_ values evidence the strong interaction between cation and the HB molecules. The main component of the complex formation energy is the interaction between the cation and the *π* electrons (cation–*π* interaction). To evidence this claim, the analysis of the electronic structure of the HCB···Na^+^ complex and of the pure HCB molecule was performed. Particularly, the energies of π molecular orbitals (MO) were compared. In analogy to the electronic structure of benzene, six *p*_z_ electrons of the C atoms form six *π* MOs (three bonding, *π*1—*π*3, and three antibonding, *π*4—*π*6) [64]. Table 4 collects the results for the isolated HCB molecule and the HCB···Na^+^ complex. Evidently, the interaction of the HCB molecule with the Na^+^ cation strongly stabilized the *π* MO orbitals by shifting their MO energies by about 4 eV. This observation confirms the strong cation–*π* interaction. Moreover, the degeneracy of bonding *π*2/*π*3 and antibonding *π*4/*π*5 MOs remained preserved in the HCB···Na^+^ complex, too.

**TABLE 4:** *π* molecular orbital energies (in eV) for HCB and HCB···Na^+^ species calculated at the PBE‐D3/def2‐TZVP level.

Molecule	*π*1	*π*2	*π*3	*π*4	*π*5	*π*6
HCB	−11.69	−6.41	−6.41	−2.41	−2.41	0.94
HCB ···Na^+^	−15.67	−10.21	−10.21	−6.40	−6.40	−3.01

**FIGURE 5:**
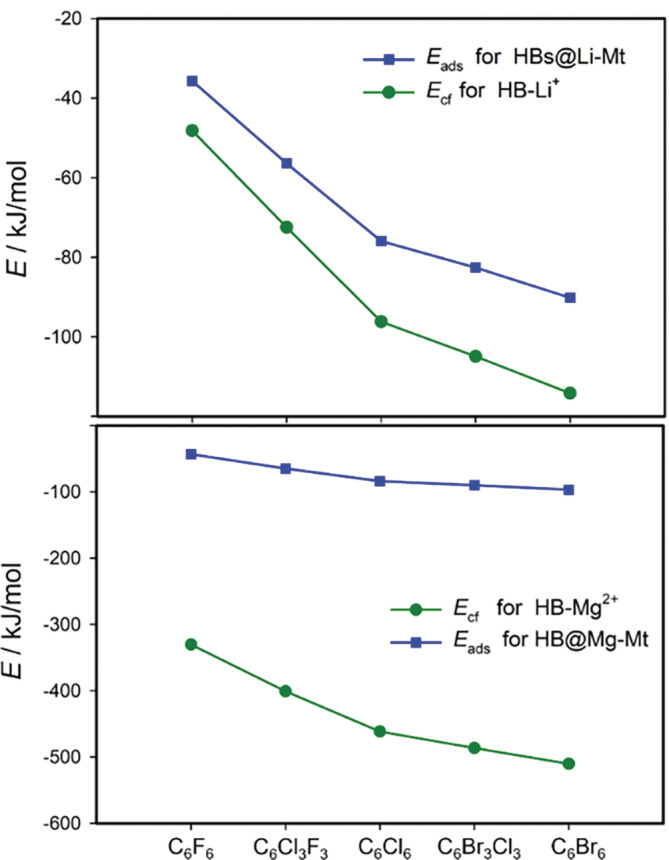
Dependences of adsorption energies (*E*_ads_) for HB···Li^+^/Mg^2+^‐Mt complexes and complex formation energies (*E*_cf_) for HB···Li^+^/Mg^2+^ gas phase complexes on HB molecular type.

Calculated *E*_cf_ decreased monotonically (in absolute value) from the smallest cations (Li^+^/Mg^2+^) to the largest cations (Cs^+^/Ba^2+^). This trend is very similar for all HB molecules. *E*_cf_ energies are larger (in absolute value) compared with the adsorption energies (*E*_ads_) for both types of cations (M(I) and M(II)). Exception is Na^+^, where *E*_cf_ are smaller than the corresponding *E*_ads_ (in absolute values) what can be a consequence of an optimal Na^+^ position between the HB molecule and the basal oxygen atoms of the Mt layer surrounding the ditrigonal hole in the adsorption HB···Na^+^‐Mt complex. The differences in *E*_ads_ and *E*_cf_ for the divalent cations are evidently larger than for the monovalent cations (Table 3 and Figure 5). Moreover, the complex formation energies for the M(II) cations are much higher (in absolute values) than for the M(I) cations (Table 3 and Figure 5). For example, *E*_cf_ for C_6_Br_6_···Mg^2+^ complex (−510.33 kJ/mol) is about five times larger than for the C_6_Br_6_···Li^+^ complex (−116.81 kJ/mol). It is not surprising as the divalent cations polarize the π‐system of the HB molecules much more than the monovalent cations.

**FIGURE 6:**
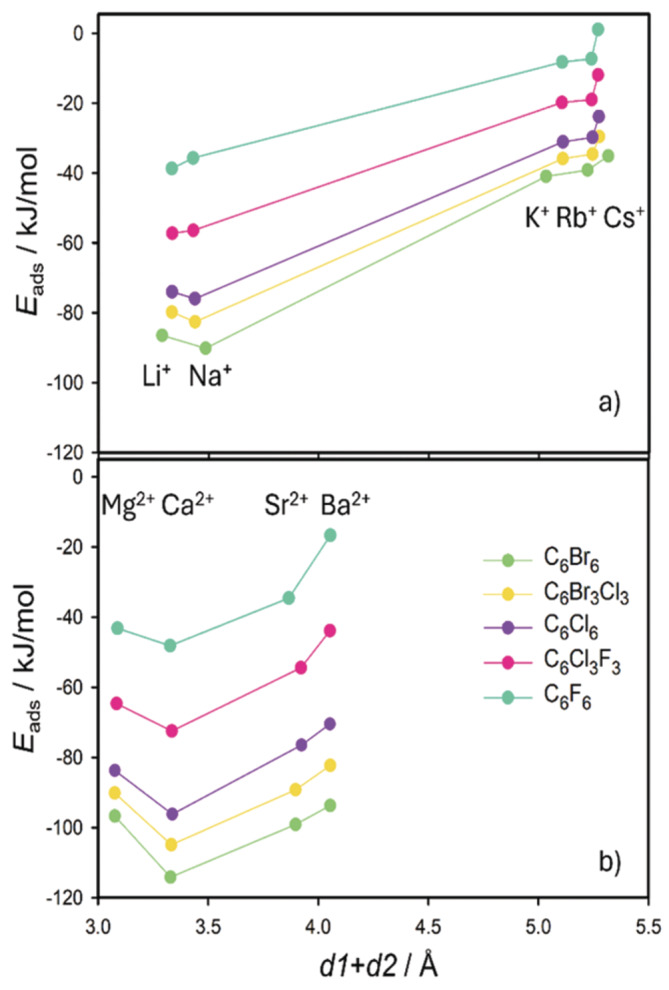
Dependences of adsorption energies (*E*_ads_) on overall perpendicular distance of the HB molecule to the plane of basal oxygen atoms (*d1* + *d2*) in all HB···M(I)/M(II)‐Mt adsorption complexes.

Further correlation was done between adsorption energies and the overall distance of the HB molecule to the plane of the basal oxygen atoms (*d1* + *d2*). Figure 6 shows the tendencies in *E*_ads_ for all five HB molecules adsorbed on the M(I)‐Mt surfaces (upper plot in Figure 6) and on the M(II)‐Mt surfaces (bottom plot in Figure 6). The separation of the M(I) cations of small and large sizes is also evident from the *E*_ads_ dependencies on the *d1* + *d2* distances. Adsorption energies are significantly larger (in absolute value) for the small Li^+^ and Na^+^ cations comparing to the three others. Some separation is also visible for the M(II) cations (Mg^2+^/Ca^2+^ vs Sr^2+^/Ba^2+^) but the difference between the strongest (Ca^2+^) and the weakest adsorption (Ba^2+^) is not as big as for the M(I) cations (Na^+^ vs Cs^+^). The trend of the adsorption energies is not monotonical going from the smallest to the largest cation. Evidently, the strongest adsorption is for Na^+^ and Ca^2+^ cations. Both cations have a similar ionic radius (about 1 Å, Table 2) and their size is “optimal” with respect to the aromatic ring size and the size of the ditrigonal hole in the Mt layer. The small cations attract HB molecules strongly resulting in the short *d1*_g_ distances in the gas phase complexes, but this distance is not optimal for the HB molecules as they would be too close to the plane of the basal oxygen atoms. It means that a repulsion force between the HB molecule and the plane of the basal oxygen atoms is prevailing over the attractive force and the HB molecule is pushed away from the surface resulting in larger *d1* distance.

**FIGURE 7:**
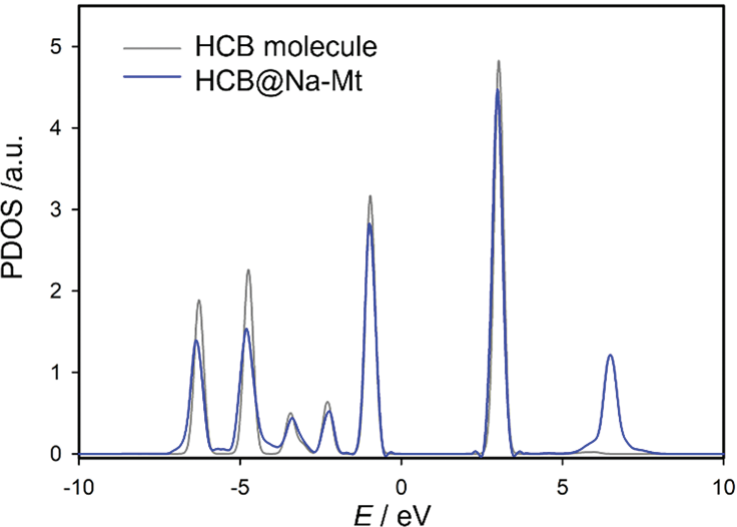
Calculated partial electronic density of states (PDOS) for *p* electrons of C atoms forming *π* ring system for isolated HCB molecule and HCB molecule in HCB···Na^+^‐Mt complex.

The driving force in the interaction of the HB molecules in the gas phase HB···M(I)/M(II) complexes is the cation–*π* interaction. However, in the adsorption complexes, the HB molecule is pushed away from the cation what can result in the weakening of the cation–*π* interaction and the increasing of the attractive dispersion interaction between the HB molecule and the basal surface oxygen atoms of the Mt layer. To show how the cation–*π* interaction and the dispersion attraction are distributed in the calculated adsorption energy, two steps were performed for the HCB···Na^+^‐Mt model. First, the projected electronic density of states (PDOS) was calculated for the *p* electrons forming the *π* ring, and, second, the adsorption energy was split on pure PBE and empirical D3 dispersion contributions. PDOS were used because the adsorption systems were calculated as models under periodic boundary conditions. The PDOS spectra of the *π* electrons is shown in Figure 7 for the isolated HCB molecule and HCB···Na^+^‐Mt model. Observed is only a very small shift to lower values of the peaks of the PVDOS spectrum in the adsorption complex comparing to the pure HCB molecule evidencing smaller cation–*π* effect comparing to the gas phase complexes. This observation is also in a relation to the separation of the adsorption energy to PBE and D3 components, shown in Table 5. Evidently, the dispersion energy is the dominant component of *E*_ads_. For the divalent cations, the D3 contribution is about 66% or more. For monovalent cations, the contribution of D3 is even greater (85% and more). In the case of the smallest cations, Li^+^ and Mg^2+^, the PBE energy is positive due to the fact that the energy analysis is for the PBE‐D3 geometry. Overall, the dominant force contributing to the adsorption energy is the dispersion force, while the cation–*π* interaction is less important. This observation is in the accordance with the previous PBE‐D3 works on the adsorption of nonpolar polycyclic aromatic hydrocarbons on the iron oxyhydroxides (FeOOH) [65], and on the adsorption of chlorinated ethenes on the mineral roaldite (γ'‐Fe_4_N) [66].

**TABLE 5:** PBE and D3 components of the adsorption energy, *E*_ads_ (in Table 3), for the HCB molecule in the HCB···Na^+^‐Mt complex in the PBE‐D3 optimized geometry. Energies are in kJ/mol.

M(I)/M/II)	Li	Na	K	Rb	Cs	Mg	Ca	Sr	Ba
$E_{\mathrm{ads}}^{\mathrm{PBE}}$	1.75	−1.76	−4.36	−4.12	−0.91	8.05	−20.89	−26.34	−23.52
$E_{\mathrm{ads}}^{D3}$	−75.74	−74.17	−26.71	−25.63	−22.92	−91.77	−75.30	−50.14	−46.97

**FIGURE 8:**
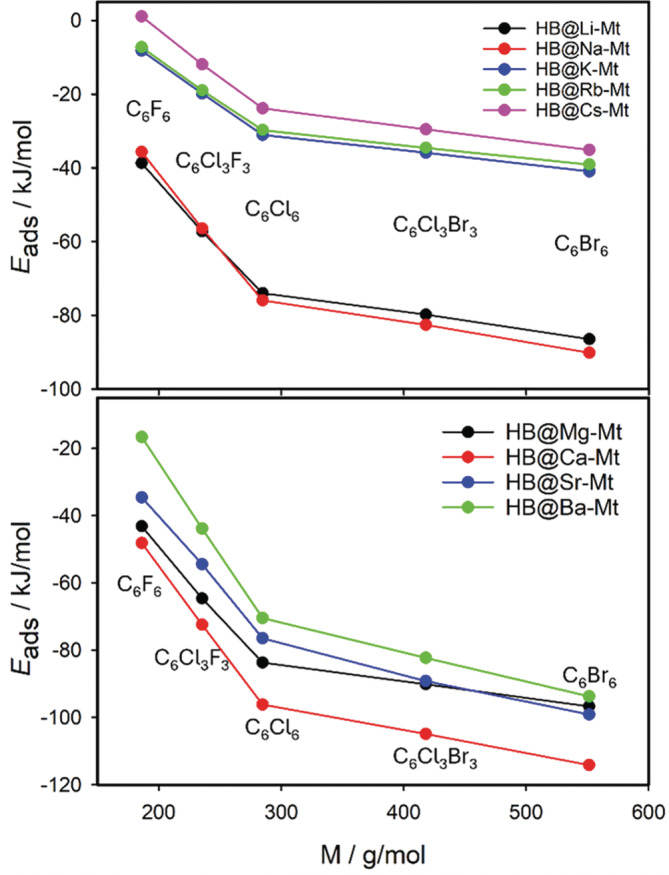
Dependences of adsorption energies (*E*_ads_) for all HB···M(I)/M(II)‐Mt complexes on the molecular mass of five HB molecules.

The final adsorption energy and the position of the HB molecules is the interplay between these two dominant forces and the ratio between them is regulated by the size of the cation (*IR*) and its charge, and by the molecular size of the HB molecule (particularly the size of the halogen atom). The trend in the adsorption energy with respect to the type of the HB molecules is the same for M(I) and M(II) cations. The strength of the adsorption is as C_6_F_6_ < C_6_Cl_3_F_3_ < C_6_Cl_6_ < C_6_Br_3_Cl_3_ < C_6_Br_6_. This is nicely visible in Figure 8 where *E*_ads_ is plotted with respect to the molecular mass (M) of the HB molecules. The largest dispersion correction was found for the largest HB molecule with the large Br atoms.

**FIGURE 9:**
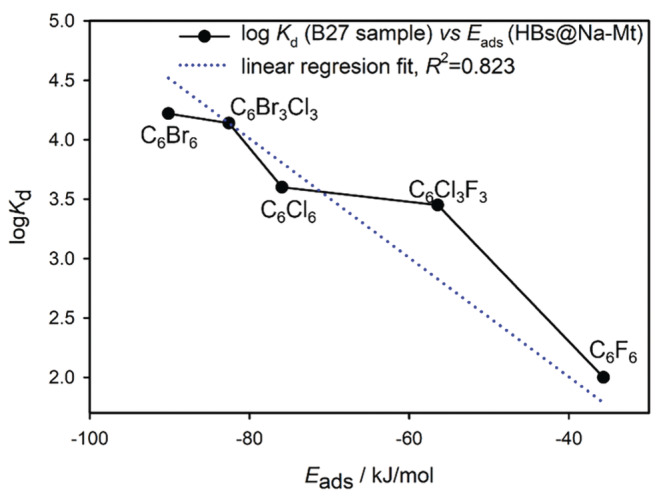
Correlation of experimentally determined solid–liquid adsorption coefficients *K*_d_ (bentonite B27) with adsorption energies (*E*_ads_) for all HB···Na‐Mt complexes.

The presented theoretical data can be referred to a gas‐phase adsorption process not including polar environment that is present in the experimental adsorption from aqueous solvent. As it was shown in our previous paper on HCB adsorption [31], the strong hydration of compensating M(I)/M(II) cations can dramatically change the strength of the adsorption. Specifically for alkali cations an increasing adsorption was observed from Li^+^ to Cs^+^ what is an opposing trend comparing to the theoretical gas‐phase adsorption energies calculated in this work (Table 3). This evidences that in modeling of adsorption process it is necessary to include solvent effect in the calculation. Moreover, also a form of the surface complex of the compensating cation (inner or outer sphere type) can play a role in the adsorption of the HBs molecules from solution. However, if we compare experimental solid–liquid adsorption data and calculated gas‐phase *E*_ads_ for the same type of the compensating cation and all five HB molecules, a good correlation is observed (Figure 9). Experimental adsorption coefficients, *K*_d_, (obtained from the fit of Henry adsorption isotherm to experimental data) were achieved for the B27 bentonite with relevant content of Na‐Mt (similar to the Wyoming Mt CMS Swy‐1–3), and details on the experiment can be found in Böhm et al. [67]. The deviations of the experimental data from the trendline in Figure 9 can be explained by the fact, that our models represent the gas phase adsorption on the ideal (001) Mt surface while the experiments were performed from the liquid phase with the real bentonite material that is not 100% montmorillonite. In addition, the bentonite sorbent is in the form of microparticles that have different surfaces available for the adsorption, (although the (001) Mt surface is dominant). Moreover, the size of pores may also affect the adsorption of HBs in a different way, since these molecules differ in molecular volume.

## 4 Conclusions

In this work, we performed a theoretical study on the adsorption of five halogenated benzenes (HBs) on the basal (001) surface of one‐layer montmorillonite models, in which the isomorphic Al^3+^/Mg^2+^ substitutions were compensated with a set of cations of alkali (Li^+^, Na^+^, K^+^, Rb^+^, Cs^+^) and alkali earth (Mg^2+^, Ca^2+^, Sr^2+^, Ba^2+^) elements. The results were compared with the results achieved for the corresponding gas phase HB···cation complexes. Energetics (adsorption energies, *E*_ads_, and gas complexation energies, *E*_cf_), and main geometrical parameters (perpendicular distances of the plane of the HB molecule to the cation and to the plane of the basal oxygen atoms) were evaluated.

The results showed how the type of the compensating cation affects the strength of the adsorption (as a measure of the adsorption energy with respect to cation radius, *IR*). The results showed that the adsorption decreases from the smallest to the largest cation for both M(I) and M(II) elements and the adsorption is stronger for alkaline earth cations comparing to alkali cations. This trend is not monotonic, the strongest adsorption was found for Na^+^ and Ca^2+^ cations, which have a similar *IR* of about 1 Å. For the five HB molecules, the strongest adsorption was observed for hexabromobenzene while the weakest adsorption was observed for the hexafluorobenzene. Adsorption increased systematically with the increasing molecular mass of HBs. The analysis of the molecular orbitals of the gas phase complexes showed the strong stabilization of the *π* MO orbitals confirming the strong cation–*π* effect and explaining large HBs···cation interaction energies. However, the PVDOS analysis and the contributions to the adsorption energy showed that the dominant component is represented by dispersion forces between the HB molecule and the basal oxygen atoms of the Mt surface and the cation–*π* interactions between *π* electrons of the aromatic ring and electron deficient compensating cation is less important. The comparison with the corresponding gas phase complexes showed that there is an interplay between the size of the HB molecule (dictated by halogen type) and the cation type (represented by its size and charge). Comparison with available experimental data for all five HB molecules adsorbed on bentonite with the high content of Na‐Mt showed a good correlation between adsorption coefficients and calculated adsorption energies. The theoretical findings of this work could be useful for the preparation of optimal adsorption materials based on montmorillonites with proper exchangeable cations.

## Data Availability

The data from DFT calculations underlying this study are openly available in Zenodo at DOI: 10.5281/zenodo.14640579. Other data will be made available upon request.
